# Apoplexy Succeeding a Long-Continued and Immoderate Use of Opium, with the Appearances on Dissection

**Published:** 1817-01

**Authors:** 


### *Apoplexy succeeding a long-continued and immoderate Use of Opium, with the Appearances on Dissection.*

By Dr. Johnson.

Mr. ——, ætat. 70, had, for several years, been afflicted with a stone in the bladder; to relieve the pain of which, he had recourse to opium. The dose had been gradually increased to *eighty grains a day!* Wishing to have greater immunity from pain than usual, he one day, about the middle of November, 1816, took ninety grains; and the next was seized with apoplexy, of which he died. Dr. Johnson assisted in opening him, twenty-four hours after death. The dura mater adhered most firmly to the skull. There was much dissolved and black blood in the sinuses. A warty excrescence was found on the left hemisphere, close to the longitudinal sinus, but of no great size: it was partly composed of a calcareous deposition. Another deposition was found near it. The vessels of the pia mater were injected with black blood. There was an effusion of water (about two ounces) in the lateral ventricles. Marks of previous and long-contiued inflammation were evident throughout the membranes lining the base of the skull, and between the convolutions of the brain. An effusion of black blood had taken place into the third ventricle (about an ounce and a half), which burst the natural parietes of that cavity, and diffused itself into the substance of the left lobe of the cerebellum. The fat was three inches deep on the abdomen. The kidnies were diseased, and in one of them an hydatid and a small calculus. The bladder contained a mulberry calculus, an ounce in weight. The inner coat of the bladder was much diseased, aud there was a small sac where the calculus had probably once lodged, when of a smaller size. There was no morbid appearance in the liver or other abdominal viscera.

